# Corticotomy-assisted orthodontics

**DOI:** 10.4317/jced.50642

**Published:** 2012-02-01

**Authors:** Jorge Cano, Julián Campo, Elena Bonilla, César Colmenero

**Affiliations:** 1 DDS, MSc, PhD. Lecturer. Department of Buccofacial Medicine and Surgery. School of Dentistry, Complutense University of Madrid. Spain; 2 DDS, PhD. Lecturer. Department of Buccofacial Medicine and Surgery. School of Dentistry, Complutense University of Madrid. Spain; 3 DDS, MSc. Orthodontist. Private practice. Madrid; 4 MD, PhD. Maxillofacial surgeon. Private practice. Madrid

## Abstract

The use of orthodontic treatment in adult patients is becoming more common and these patients have different requirements specially regarding duration of treatment and facial and dental aesthetics. Alveolar corticotomy is an effective means of accelerating orthodontic treatment. This literature revision include an historical background, biological and orthodontic fundamentals and the most significant clinical applications of this technique. Orthodontic treatment time is reduced with this technique to one-third of that in conventional orthodontics. Alveolar bone grafting of labial and palatal/lingual surfaces ensures root coverage as the dental arch is expanded. Corticotomy-assisted orthodontics has been reported in a few clinical cases, and seems to be a promising adjuvant technique, indicated for many situations in the orthodontic treatment of adults without active periodontal pathology. Its main advantages are reduction of treatment time and postorthodontic stability. Further controlled prospective and histological studies are needed to study tooth movement, post-retention stability, and microstructural features of teeth, periodontium, and regenerated bone after using this procedure.

** Key words:**Corticotomy, osteotomy, accelerated orthodontics.

## Introduction

The use of orthodontic treatment in adult patients is becoming more common. These patients have different requirements regarding duration of treatment, concerns regarding facial and dental aesthetics, and types of appliance that can be used. Additionally, orthodontic treatment in adult patients has special features with regard to periodontal hyalinization and alveolar flexibility compared with growing patients (1).

Corticotomy-assisted or corticotomy-facilitated orthodontics is a therapeutic procedure that helps orthodontic tooth movement by accelerated bone metabolism due to controlled surgical damage. This is not a new procedure, although it was initially based more on techniques using osteotomy instead of approaches with corticotomy. It is considered an intermediate therapy between orthognatic surgery and conventional orthodontics. The procedure has several advantages, such as reduced treatment time and facilitation of dental arch expansion. It also makes possible differential tooth movement (i.e., impacted teeth) and shows improved post-orthodontic stability (1,2).

This article presents a review of the literature, including the historical background, biological and orthodontic fundamentals, based on experimental studies, and the most significant clinical applications, based on recently published clinical studies.

## Historical Background

In 1893, Cunningham presented “Luxation, or the immediate method in the treatment of irregular teeth” at the International Dental Congress in Chicago. He used mesial and distal interseptal osteotomies with a circular saw to reposition palatally inclined maxillary teeth and stabilized them in correct occlusion with wire ligatures or metal splints for 35 days. The most important feature was the fact that this combined active surgical-orthodontic treatment reduced the procedure time to one-third that of conventional treatment and allowed more predictable treatment in older patients. Fifty years later, Bichlmayr classified orthognathic surgery as “major” (total or segmental maxillary and mandibular correction) or “minor” (interdental osteotomy or corticotomy), and was the first to described the corticotomy procedure to close diastemata in patients over 16 years old. This procedure was used to correct maxillary incisor protrusion by extraction of the first premolars, division of the palatal cortex overlying the incisors, and excision of alveolar bone distal to the canines.

After these initial approaches, some surgeons combined both procedures (alveolar osteotomy and corticotomy) to reduce the duration of orthodontic treatment. Köle (1) popularized the procedure in the English literature with his “bony block” technique. He reported some cases in which interdental vertical corticotomy and subapical horizontal osteotomy were combined dividing the alveolar process in its entirety apical to the ends of the roots, correcting retrusive (i.e., deep overbite) or protrusive incisors (i.e., open bite or diastemic incisors). He also reported buccal corticectomy in posterior inferior sectors to correct molar linguoversion and facilitate orthodontic expansion. He relied on the reduction of cortical resistance and allowing vascular supply from the trabecular bone to the teeth some years before the vascular supply of alveolar maxillary bone was described by Bell (2). Buccal and palatal corticotomies have also been described to correct compressed maxilla to improve the alveolar expansion and limit the buccal tilting of the posterior teeth (1,3).

Bell and Levy(4) published the first experimental study of alveolar corticotomy in 49 monkeys in 1972. They described a model of vertical interdental corticotomy that should have been considered an osteotomy, because they mobilized all dento-osseus segments. Additionally, they performed reflection of labial and palatal flaps simultaneously, which markedly compromised the blood supply to the anterior teeth. A histological study showed the risk of this type of procedure (full mucoperiosteal detachment plus cutting of medullar bone) for the vascularity of dental pulp and surrounding medullar bone. They demonstrated distinct avascular zones that progressively recovered after 3 weeks postoperatively, except for the central incisors.

Duker (5) investigated how corticotomy affected the vitality of the teeth and the marginal periodontium in beagle dogs. Rearrangement of the teeth within a short time after corticotomy damaged neither the pulp nor the periodontal ligament (PDL). He supported the idea of preserving the marginal crest bone in relation to interdental cuts; these cuts must always be left at least 2 mm short of the alveolar crestal bone level.

These initial approaches included some types of alveolar osteotomy alone or combined with corticotomy, called “bone block movement.” Traditionally, vertical and horizontal osteotomies have had an increased risk of postoperative tooth devitalization or even bone necrosis, depending on the severity of injury to the trabecular bone. There is also an increased risk of periodontal damage, mainly in cases in which the interradicular space is less than 2 mm (6). Corticotomy has many advantages compared with osteotomy. It prevents injury of the periodontium and pocket formation, and also prevents devitalizing of a single tooth or a group of teeth. The nutritive function of the bone is maintained through the spongiosa, although the bone is exposed, avoiding the possibility of bone aseptic necrosis(1).

Taking into account these advantages and drawbacks, procedures based on corticotomy are gradually replacing those using osteotomy. Wilcko et al.(7,8) described an innovative strategy of combining corticotomy alveolar surgery with alveolar grafting in a technique referred to initially as accelerated osteogenic orthodontics (AOO) and more recently as periodontally accelerated osteogenic orthodontics (PAOO). This technique combines fixed orthodontic appliances, labial and palatal/lingual corticotomies, and bone grafting with demineralized freeze-dried bone and bovine bone with clindamycin. Tooth movement was initiated 2 weeks after surgery, and every 2 weeks thereafter by activation of the orthodontic appliance. Wilcko et al. (7,8) were first to suggest that tooth movement assisted with corticotomy may be due to a demineralization-remineralization process rather than bony block movement.

## Biological and orthodontic fundamentals

It is important to state the differences between the four types of surgical damage in alveolar bone: Osteotomy (complete cut through cortical and medullar bone), corticotomy (partial cut of cortical plate without penetrating medullar bone), ostectomy (removal of an amount of cortical and medullar bone) and corticotectomy (removal of an amount of cortex without medullar bone).

One of the main disadvantages of conventional orthodontic treatment is time, requiring more than 1 year for completion. There are three options to shorten the time of treatment: (i) local administration of chemical substances, (ii) physical stimulation (i.e., electrical current or magnets), and (iii) surgery (i.e., alveolar corticotomy, compression, or distraction). Corticotomy-assisted orthodontics (CAO) has been employed to speed up orthodontic treatment. Some clinical studies have shown a reduction of treatment time by one-third compared with conventional treatments. CAO has additional advantages, such as less root resorption, due to decreased resistance of cortical bone, more bone surrounding teeth, due to addition of bone graft, less and slower relapse, and less need for extraoral appliances and orthognathic procedures (7,8).

The regional acceleratory phenomenon (RAP) is a local response of tissues to noxious stimuli by which tissue regenerates faster than normal (i.e., without stimuli) in a regional regeneration/remodeling process(9). This is an intensified bone response (increased osteoclastic and osteoblastic activity, and increased levels of local and systemic inflammation markers) in areas around cuts that extend to the marrow. This response varies directly in duration, size, and intensity with the magnitude of the stimulus, and it is considered a physiological “emergency” mechanism, which accelerates the healing of injuries that could affect survival. The duration of RAP depends on the type of tissue, and usually lasts about 4 months in human bone. This phenomenon causes bone healing to occur 10–50 times faster than normal bone turnover(9).

The healing phases of RAP have been studied in the rat tibia. There is an initial stage of woven bone formation, which begins in the periosteal area and then extents to medullar bone, reaching its maximal thickness on day 7. This cortical bridge of woven bone is a fundamental component of RAP, providing mechanical stability of bone after injury. From day 7, the woven bone in the cortical area begins to undergo remodeling to lamellar bone, but woven bone in the medullary area undergoes resorption, which means transitory local osteopenia. It seems that medullar bone needs to be reorganized and rebuilt after establishment of the new structure of cortical bone, and to adapt to the reestablishment of cortical integrity (3 weeks in rats). There is also a systemic acceleratory phenomenon (SAP) of osteogenesis due to systemic release of humoral factors(10). In human long bones, RAP begins within a few days after surgery, usually peaks at 1-2 months, and may take from 6 to 24 months to subside completely(11).

Clinically, alveolar bone exposure after reflection of soft tissue flaps is known to cause some degree of bone resorption, mainly around teeth or dental implants. This RAP has been observed not only after corticotomy of the alveolar bone but also after full-thickness mucoperiosteal flap rejection without touching the bone. A study in the mandibles of rats showed transitory trabecular bone resorption after flap reflection. The degree of resorption was greater if lingual and buccal flaps were used, was greater in the lingual plate, and peaked with maximum resorption at 3 weeks after surgery, which is equivalent to 3 months in humans. The alveolar bone recovered to control levels at 120 days after surgery. This may be responsible for the increased tooth mobility after periodontal surgery(12).

Surgical injury causes transient osteopenia in alveolar bone (i.e., a temporal and reversible decrease in bone mineral density)(13). This reduces the biomechanical resistance and enables rapid tooth movement through trabecular bone. Transient osteopenia may be prolonged with loading orthodontic application, taking into account that we have a limited spatiotemporal window that limits the RAP to the teeth surrounded by corticotomy over a range of time (estimated 3–4 months). This is why it is imperative to adjust the orthodontic appliance every 2 weeks(14).

There is an increase in tooth mobility during CAO treatment due to the transient osteopenia without a change in bone matrix volume(14,15). It is generally accepted that heavier forces must be applied in cases of “bone block” movement after corticotomy to move the tooth–bone block(1,3). However, it has been reported that conventional orthodontic forces are sufficient in CAO because forces are not concentrated in either the tooth–periodontal complex surrounded by a rigid bone structure or in the bone-tooth block delimitated by corticotomy, but is distributed on the tooth–periodontal–trabecular bone (low-density transitory trabecular bone). This better distributed mechanical loading may be the reason why corticotomy-assisted tooth movement is associated with a reduced period of PDL hyalinization on the compression side compared to conventional movement (1 week instead of 4 weeks in the beagle dog mandible). This prolonged period of hyalinization may explain some degree of root resorption in conventional movement, which is not observed in CAO(16,17).

Hyalinization (tissue necrosis) is caused by excessive compression of the PDL as a result of excessive pressure, which suppresses blood supply, although it may appear even with light force(18,19). This hyalinized tissue attracts neutrophil granulocytes and macrophages by chemotaxis and must be removed and remodeled before starting bone resorption by osteoclasts and subsequent orthodontic tooth displacement. Vascular access of osteoclasts to the PDL–lamina–dura interface is limited when the PDL is compressed. Thus, extensive and prolonged hyalinization of the PDL results in slower tooth movement. After Reitan’s studies (20), this period of hyalinization has been designated as the lag phase or arrest phase of orthodontic tooth movement, after the initial phase and before the acceleration or post-lag phase.

In a study performed in dogs, Von Böhl et al.(21) reported that these hyalinized areas appear not only between 4 and 20 days but also in the acceleration phase (40–80 days of tooth movement) as a continuous process. The absence of this necrotic tissue leads to direct bone resorption and faster tooth movement. However, the rate of tooth movement, which is influenced by hyalinization, is not only directly related to force magnitude, but also to the type of movement (bodily tooth or tipping movement) or the individual bone metabolic capacity (bone density, systemic and genetic factors)(19).

Traditionally, bone distraction in long bones with corticotomy or osteotomy has been reported to show similar results. However, craniofacial bones have a medullar bone with a different macrostructure than long bones. Lee et a l(22) reported the differences in bone regeneration after corticotomy and osteotomy when orthodontic forces were applied in the maxillae of rats as a model system. By micro-computed tomography (micro-CT), they found that osteotomy areas resembled regeneration by distraction, while corticotomy areas showed a regional loss of bone supporting teeth (radiological demineralization), typical of RAP. It seems that corticotomy opens the underlying marrow vascular spaces, enhancing healing potential, but maintaining the segment in a stable state and creating a demineralized region. Bone block movement after osteotomy creates a dynamic microenvironment similar to distraction osteogenesis, but does not show regional demineralization in medullar bone. They proposed that the mechanism of tooth movement is likely to be different for those teeth included in corticotomy or osteotomy. This research group described differences in molecular biology between the groups (corticotomy and osteotomy). The group with corticotomy and tooth movement showed three phases of bone healing: a resorptive phase on day 3 (more osteoclasts), a replacement phase on day 21 (more osteoblast-like cells), and a mineralization phase on day 60 (non-lamellar bone formation) in the compression site. The osteotomy and tooth movement group was quite different, and had no bone resorption or replacement phase, but showed progressive bone formation and an increased number of vessels in sites distal from the osteotomy, resembling a distraction process(23).

Some root resorption is generally expected with any orthodontic tooth movement, and its extent depends on the duration of force application. Ren et al.(17) reported rapid tooth movement after CAO in beagles without any associated severe root resorption or irreversible pulp damage. Some mild root resorption was observed after 4 weeks, which was partially repaired by week 8.

It has also been reported in beagle dogs, that corticotomy allows a greater degree of tooth movement, fourfold larger in the maxilla and two times that in the mandible, compared to conventional orthodontic movement. Teeth with cortical activation also show greater cellular activity. Osteoclasts, fibroblasts, cementoblasts, and osteoblasts showed greater cellular activity in the PDL and on both the tooth and bone surface. This increased cellular activity decreased after 8 weeks, and after a further 6 months this cellularity decreased markedly and bone matrix became denser and more mature at extended time points after cortical activation(24) .

Another histological study in rats showed that there was increased turnover of alveolar spongiosa immediately adjacent to the decortication areas, without any orthodontic force being applied. Trabecular bone surface area decreased by half and PDL surface area increased by twofold. Levels of catabolic and anabolic activity were three times greater at 3 weeks after surgery, and these increases decreased gradually until the 7th week, then reached a steady-state by the 11th week after surgery(15).

## Clinical Applications

The CAO procedure has been reported to solve some clinical situations difficult to treat by conventional orthodontic means, including resolution of tooth crowding, shortening of treatment time, canine retraction after premolar extraction, facilitation of impacted tooth eruption, facilitation of slow orthodontic expansion, molar intrusion with open bite correction, and enhancement of postorthodontic stability(25).

There are a number of clinical principles that must be used to guide this procedure.

The orthodontist determines the plan for movement, identifying which tooth or temporary device will be used for anchorage and which teeth require corticotomy (orthodontist–surgeon communication).

Periodontal view of the surgery, using minimally invasive incisions and flaps, taking care to preserve the papillae.

The purpose of the decortications is to initiate the RAP response and not to create movable bone segments.

Initiation of orthodontic force should not be delayed by more than 2 weeks after surgery.

Orthodontic clinical appointments must be closer together (2 weeks) because of the faster rate of tooth movement.

The orthodontist has a “window” period for accelerated movement (4–6 months) after which movements occur at normal speed(26).

Wilcko et al(8). reported two adult patients with severe crowding who were treated by CAO in just 6.5 months. Wilcko et al. (7) also reported a case in an adult woman who was treated in 7 months, with occlusion that remains stable at 8 years of retention.

Treatment of maxillary constriction with slow palatal expansion may include a risk of removing tooth roots from their bone envelope. The addition of CAO has been reported clinically to increase stability and improve periodontal health. Additionally, unilateral expansion may be better controlled by unilateral corticotomy on the crossbite side, without unnecessary overexpansion on the normal side(25). Lateral expansion of such constricted cases may cause significant bony dehiscence or fenestration. Wilcko et al.(8) described a case where labial and lingual root prominences were no longer evident after CAO and graft procedure after 2.5 years of retention. Bone biopsy after the consolidation period indicated lamellar bone in these areas.

Since the first reports of the procedure, there have been published cases of severe anterior open bite where corticotomy in the anterior alveolar bone allowed extrusion movement of anterior teeth, observing stability after a 1-year follow-up(27). There are side effects of any orthodontic mechanics unless we use a temporary skeletal anchorage device (microscrews, mini-implants, miniplates). These devices are considered a dramatic step forward in the control of complex orthodontic movement. Yao et al.(28) described molar intrusion of 4 mm in 7.6 months using these temporary devices. On the other hand, the same procedure combined with CAO can achieve the same amount of intrusion in 2.5 months(29). However, temporary anchorage devices cannot always be used due to anatomical or financial reasons, or they may not be able to achieve the desired results by themselves. There have been reports of some complex movements were CAO was very helpful. For example, intrusion of the upper molars for prosthodontic reasons combining buccal corticotectomies with microscrews or conventional straight arch wires; or intrusion of upper molars to close an anterior open bite with oblique headgear(13). Another indication for CAO is intrusion of molars for prosthodontic reasons. These cases require some corticotectomy, depending on the amount of intrusion. Miniplates and microscrews are used with buccal and palatal elastic traction of 100–150 grams (g) per side, observing the results after 2 months (3 mm of intrusion). This procedure may be designated as compression osteogenesis (CO) instead of CAO, because the bone–tooth block is only supported by medullar bone and overlying mucosa and it has lost all of its cortical support(30).

Cases of bimaxillary protrusion can be retracted with premolar extraction and conventional fixed appliances. However, cortical palatal plates may limit this movement at the level of the incisor apices, even when premolar extractions have been done. A horizontal palatal corticotectomy behind the upper incisor and a horizontal labial corticotomy may resolve this situation (two stages). In this situation, there is a block of bone (pedicled with medullary bone) that must be moved rather than moving teeth through the bone, which is another model of the CO procedure. Thus, a heavier orthopedic force of 500–900 g must be applied by anchorage of palatal bone plates(31). This concept of compression osteogenesis uses similar biological fundamentals to CAO, but with corticotectomy instead of corticotomy. Thus, movement of the bone block with included teeth in CO is larger and more unstable than movement of teeth in the weakened alveolar bone of CAO. The resulting structure in CO simulates a “floating bone” model where the medullary bone and overlying soft tissue are the pedicle for the teeth where the orthodontic forces are applied.

Stability after treatment has always been an important concern after orthodontic treatment. Thinner mandibular cortices are a risk feature for bony dehiscence after decrowding orthodontic treatment. Techniques that increase alveolar volume with grafts may resolve this situation; thus, where 5 mm of crowding was considered the limit for traditional orthodontics without extraction, this may be extended to 10–12 mm without risk of dehiscence. Retention and stability with CAO are better that with conventional orthodontics (32). However, there have been no long-term prospective longitudinal studies supporting these initial results.

Kanno et al.(33) described a CO procedure used to treat a case of severe open bite, moving the upper posterior bone–tooth segments 7 mm in a superior position. They used anchor plates and elastics 3 weeks after surgical intervention in two stages. Satisfactory results were obtained after 6 months of orthodontic treatment.

Variations of the initial technique have been reported as less invasive and to have less swelling and hematoma after surgery. Dibart et al.(14) described a tunnel approach with piezoelectric bone cuts. Several vertical inci-sions are distributed on the attached gingiva through piezoelectric vertical corticotomies. The tunneling approach allows placement of the bone graft. A case of mild crowding was solved within 17 weeks of active treatment with this approach.

CAO is a promising adjuvant technique, indicated for many situations in the orthodontic treatment of adults. It has been used in some limited cases to avoid secondary effects of conventional orthodontics, such as root re-sorption in molar intrusion or periodontal dehiscence in slow tooth expansion. However, its main advantages are reduction of treatment time and postorthodontic stability, which may allow its generalized use in many adult patients without active periodontal pathology. The biological principle of this method is based on temporary reduction of medullar bone density (transitory osteopenia) within a 3–4-month window, which allows more physiological tooth movement inside the alveolar bone (less hyalinization period of PDL).

## References

[1] Kole H (1959). Surgical operations on the alveolar ridge to correct occlusal abnormalities. Oral Surg Oral Med Oral Pathol.

[2] Bell WH (1969). Revascularization and bone healing after anterior maxillary osteotomy. A study using adult rhesus monkeys. J Oral Surg.

[3] Converse JM, Horowitz SL (1969). The surgical-orthodontic approach to the treatment of dentofacial deformities. Am J Orthod.

[4] Bell WH, Levy BM (1972). Revascularization and bone healing after maxillary corticotomies. J Oral Surg.

[5] Düker J (1975). Experimental animal research into segmental alveolar movement after corticotomy. J Maxillofac Surg.

[6] Merrill RG, Pedersen GW (1976). Interdental osteotomy for immediate repositioning of dental-osseous elements. J Oral Surg.

[7] Wilcko MT, Wilcko WM, Pulver JJ, Bissada NF, Bouquot JE (2009). Accelerated osteogenic orthodontics technique: a 1-stage surgically facilitated rapid orthodontic technique with alveolar augmentation. J Oral Maxillofac Surg.

[8] Wilcko WM, Wilcko T, Bouquot JE, Ferguson DJ (2001). Rapid orthodontics with alveolar reshaping: two case reports of decrowding. Int J Periodont Restorat Dent.

[9] Frost HM (1983). The regional acceleratory phenomenon: a review. Henry Ford Hosp Med J.

[10] Schilling T, Müller M, Minne HW, Ziegler R (1998). Influence of inflammation-mediated osteopenia on the regional acceleratory phenomenon and the systemic acceleratory phenomenon during healing of a bone defect in the rat. Calcif Tissue Int.

[11] Frost HM (1989). The biology of fracture healing. An overview for clinicians. Part II. Clin Orthop Relat Res.

[12] Yaffe A, Fine N, Bindermann I (1994). Regional accelerated phenomenon in the mandible following mucoperiosteal flap surgery. J Periodontol.

[13] Oliveira DD, Franco B, Villamarin R (2010). Alveolar corticotomies in orthodontics: indications and effects on tooth movement. Dent Press J Orthod.

[14] Dibart S, Sebaoun JD, Surmenian J (2009). Piezocision: a minimally invasive, periodontally accelerated orthodontic tooth movement procedure. Compend Contin Educ Dent.

[15] Sebaoun JD, Kantarci A, Turner JW, Carvalho RS, Van Dyke TE, Ferguson DJ (2008). Modeling of trabecular bone and lamina dura following selective alveolar decortication in rats. J Periodontol.

[16] Iino S, Sakoda S, Ito G, Nishimori T, Ikeda T, Miyawaki S (2007). Acceleration of orthodontic tooth movement by alveolar corticotomy in the dog. Am J Orthod Dentofacial Orthop.

[17] Ren A, Lv T, Kang N, Zhao B, Chen Y, Bai D (2007). Rapid orthodontic tooth movement aided by alveolar surgery in beagles. Am J Orthod Dentofacial Orthop.

[18] Rygh P (1973). Ultrastructural changes in pressure zones of human periodontium to orthodontic tooth movement. Acta Odontol Scand.

[19] Pilon JJ, Kuijpers-Jagtman AM, Maltha JC (1996). Magnitude of orthodontic forces and rate of bodily tooth movement. An experimental study. Am J Orthod Dentofacial Orthop.

[20] Reitan K (1967). Clinical and histologic observations on tooth movement during and after orthodontic treatment. Am J Orthod.

[21] Von Böhl M, Maltha J, Von den Hoff H, Kuijpers-Jagtman AM (2004). Changes in the periodontal ligament after experimental tooth movement using high and low continuous forces in beagle dogs. Angle Orthod.

[22] Lee W, Karapetyan G, Moats R, Yamashita DD, Moon HB, Ferguson DJ (2008). Corticotomy-/osteotomy-assisted tooth movement microCTs differ. J Dent Res.

[23] Wang L, Lee W, Lei DL, Liu YP, Yamashita DD, Yen SL (2009). Tisssue responses in corticotomy- and osteotomy-assisted tooth movements in rats: histology and immunostaining. Am J Orthod Dentofacial Orthop.

[24] Cho KW, Cho SW, Oh CO, Ryu YK, Ohshima H, Jung HS (2007). The effect of cortical activation on orthodontic tooth movement. Oral Dis.

[25] Hassan AH, Al-Fraidi AA, Al-Saeed SH (2010). Corticotomy-assisted orthodontic treatment: review. Open Den J.

[26] Murphy KG, Wilcko MT, Wilcko WM, Ferguson DJ (2009). Periodontal accelerated osteogenic orthodontics: A description of the surgical technique. J Oral Maxillofac Surg.

[27] Generson RM, Porter JM, Stratigos GT (1978). Combined surgical and orthodontic management of anterior open bite using corticotomy. J Oral Surg.

[28] Yao CC, Wu CB, Wu HY, Kok SH, Chang HF, Chen YJ (2004). Intrusion of the overerupted upper left first and second molars by miniimplants with partial-fixed orthodontic appliances: a case report. Angle Orthod.

[29] Oliveira DD, de Oliveira BF, de Araújo Brito HH, de Souza MM, Medeiros PJ (2008). Selective alveolar corticotomy to intrude overerupted molars. Am J Orthod Dentofacial Orthop.

[30] Chung KR, Kim SH, Lee BS (2009). Speedy surgical-orthodontic treatment with temporary anchorage devices as an alternative to orthognathic surgery. Am J Orthod Dentofacial Orthop.

[31] Moon CH, Wee JU, Lee HS (2007). Intrusion of overerupted molars by corticotomy and orthodontic skeletal anchorage. Angle Orthod.

[32] Sebaoun JD, Ferguson DJ, Wilcko MT, Wilcko VM (2007). Alveolar osteotomy and rapid orthodontic treatments. Orthod Fr.

[33] Kanno T, Mitsugi M, Furuki Y, Kozato S, Ayasaka N, Mori H (2007). Corticotomy and compression osteogenesis in the posterior maxilla for treating severe anterior open bite. Int J Oral Maxillofac Surg.

